# Sex and the basic scientist: is it time to embrace Title IX?

**DOI:** 10.1186/2042-6410-4-13

**Published:** 2013-07-27

**Authors:** Kathryn Sandberg, Joseph G Verbalis

**Affiliations:** 1 Department of Medicine, Georgetown University, 4000 Reservoir Road, NW, Washington, DC 20057, USA

## Background

New drugs must be deemed safe and effective by the Food and Drug Administration (FDA) before they are made available to the public. The FDA can also withdraw drugs from the market if unexpected adverse effects are detected in postmarketing surveillance after initial approval. The U.S. General Accounting Office (GAO) reviewed ten prescription drugs withdrawn from the market during the period of January 1997 and December 2000 and found that 8 out of the 10 drugs were withdrawn due to adverse events occurring predominantly in women. Women were at greater risk for valvular heart disease for the appetite suppressants fenfluramine and dexfenfluramine, and Torsades de Pointes for the antihistamines terfenadine and astemizole [1]. In fact, the FDA filed a public health advisory in 1997 noting that valvular heart disease discovered in patients receiving fenfluramine and phentermine were all women [2] and women were found to make up 70% of adverse event reports of Torsades de Pointes that was induced by drugs that prolong cardiac repolarization [3]. The FDA also is responsible for determining indications and dosing for approved drugs. On May 14, 2013, the FDA issued a safety communication approving label changes to zolpidem-containing medications for treatment of insomnia and recommended significantly lower doses in women for immediate-release products because women are more susceptible than men to the risks posed by “next-day impairment of driving and other activities that require full alertness” [4]. This was a landmark decision by the FDA since no prior FDA labels recommended different dosages for men and women if the product was intended for use by both sexes.

Why were these sex differences discovered only after the drug was marketed? A 1992 GAO study [5] suggested that a major reason has been the lack of representation of women in clinical trials, and most likely, as a direct result of the 1977 FDA guidelines [6] that effectively excluded women of childbearing age from participating in clinical trials. Once President Clinton signed the National Institutes of Health (NIH) Revitalization Act into law in 1993, which included a mandate that the NIH ensure that women are included in all research involving human subjects, clinical research was transformed. Since then, not only government-funded, but also pharmaceutical-funded clinical trials have routinely included substantial numbers of women. Yet, even after 1993, there were more reports of unexpected adverse events in women than men during post-marketing surveillance [1]. Why?

To address this question, it is necessary to understand the phases of clinical trials that a candidate drug must pass before it is considered by the FDA for market approval. Before large Phase III/IV clinical trials are initiated to confirm the efficacy and safety of a new drug in clinical populations, potential new therapeutics are tested for safety in Phase I trials. Once a drug passes accepted safety standards, efficacy testing using quantitative outcome measures begins in Phase II; however, these Phase I and II studies are not required to compare dose and efficacy between the sexes. In fact, out of all the bioequivalence studies submitted to the Center for Drug Evaluation and Research between 1977 and 1995, 29 included data addressing sex differences in drug absorption and of those 29, only 26 met the statistical criterion for sex difference analysis [7]. Thus, the lack of comparison of dose and efficacy as a function of sex prior to the design of larger Phase III/IV clinical trials could be an explanation for why unexpected adverse events can exhibit a sex bias towards women since women are underrepresented in early phase clinical trials. For example, Pinnow et al. [8] showed that of the Phase I trials approved by the FDA between 2006 and 2007, only 32.5% of the participants were women. In an in-depth analysis of new medical entities approved by the FDA during 2000–2002, Yang et al. [9] found that although there has been overall progress in the inclusion of women in Phase III clinical trials regulated by the FDA, there was still a significant variation in female participation by class of drug (e.g., women were underrepresented in drugs targeting renal and cardiovascular disease) and there were no mandatory FDA requirements for prospectively designing clinical trials to investigate the impact of one’s sex or in conducting the appropriate and complete analyses by sex. All of these factors could contribute to sex differences in adverse events.

The study by Kwekel et al. [10] just published in this journal illustrates why sex bias in basic biomedical research provides an additional explanation for the greater occurrence of unexpected adverse events in women than men. Using whole genome microarray gene expression analysis of kidneys from male and female rats at different ages, they identified hundreds of genes (841) that are differentially expressed in a sex-specific manner at one or more ages across the life span of rats and 114 sex-biased genes (69 female-biased and 45 male-biased) that were common to all three age groups examined. These findings support previous studies demonstrating sexually dimorphic gene expression. In 2006, Yang et al. [11] performed microarrays on multiple somatic tissues of large numbers of mice derived from an intercross between inbred mouse strains, and showed that hundreds of mouse genes were expressed in the brain in a sexually dimorphic manner while thousands of genes were sexually dimorphic in liver, adipose tissue, and muscle. Van Nas et al. [12] then showed in mouse liver that sex differences in gene expression are in large part due to activational effects of gonadal hormones in adulthood, although gonadal hormone-independent sex chromosome effects also contributed to sex-biased gene expression. By showing how age impacts sex differences in the organization of renal gene expression networks, Kwekel et al. support and extend the previous study by Van Nas et al., which uncovered sex-specific gene expression networks related to genetic and metabolic traits (especially in adipose and liver tissue) as well as gonadal hormone status.

Early diagnosis of drug-induced kidney injury is a key factor in pharmaceutical safety and decision making. Of major concern is the finding by Kwekel et al. [10] that five out of the six gene products qualified by the FDA for use in preclinical monitoring of disease and drug-induced nephrotoxicity [13] exhibit major sex differences in mRNA expression including kidney injury molecule 1, clusterin, trefoil factor 3c, osteopontin and lipocalin 2. For example, kidney injury molecule 1 exhibits a 23-fold sex difference in mRNA expression at the age (8 weeks) at which *in vivo* toxicity evaluations in animals are most commonly conducted; however, biomarker performance testing that led to FDA qualification of these biomarkers were predominantly conducted in male animals [14]. Kwekel et al. also found sexual dimorphic mRNA expression of renal transporters critically involved in drug uptake and excretion (e.g., *Slco1a1, Slc22a7, Slc22a2 and Abcg2*) with two transporters that handle a major portion of prescribed drugs (i.e., *Slco1a1* and *Slc22a7*) exhibiting directly opposite sex-biased gene expression. Taken together, these findings strongly indicate significant differences in the biology of renal protein expression and urinary excretion exist between males and females. Thus, one potential reason adverse events are more common in women than men is because the choice of drug targets are male-biased as a result of the male bias in basic biomedical research (i.e., at the stage of drug target identification). While there is a growing awareness of sex differences in the pharmacokinetics and pharmacodynamics of drug action [15], basic science research is still predominantly conducted using male animals. A survey of animal research published in major journals in 2009 across ten different disciplines revealed a male bias in eight out of ten fields surveyed including neuroscience, physiology, pharmacology, endocrinology, zoology, and behavior [16]. Studies in cell culture are also predominantly conducted in XY cells even though sex-specific pathways in cell fate and mechanisms of cell death exist and play critical roles in numerous human pathologies [17].

## Discussion

Title IX is a U.S. federal law enacted in 1972 that bans sex discrimination in any federally-funded education program. The U.S. Department of Justice reported in June 2012 that Title IX by providing girls and women equal access to education, has dramatically expanded women’s access to athletic programs and increased their educational attainment [18]. Before Title IX, only one in 27 girls played in varsity high school sports, whereas by 2001 one in every 2.5 girls participated. Since 1972, the number of female athletes in college has increased by 450%. Not only are there long-lasting health benefits from engaging girls and women in athletics, women who are more active in sports have more self-confidence, team-building and leadership skills. In fact, 80% of female managers of Fortune 500 companies have a sports background. As of March 2013, National Collegiate Athletic Association scholarships virtually nonexistent before Title IX now exceed $960 million for women, which has contributed to the increase in percentage of women enrolled in college. Last year, at the 40th anniversary of Title IX, President Obama commented on many of these positive aspects including that this law “has helped to make our society more equal in general”.

President Clinton’s actions in 1993 had a landmark positive effect on reducing sex discrimination in federally funded clinical research. Is now the time to eliminate sex discrimination in federally funded basic biomedical research? We strongly feel it is for multiple reasons. First, it is extremely wasteful to develop a drug that ends up being removed from the market during the late stages of drug development because of adverse consequences in women. Second, by not investigating basic biological mechanisms in the female from the outset, we fail to understand the full biology and pathophysiology of girls and women. Third, we need to capitalize on the clues to disease that are reflected in the sex differences underlying observations like the onset of hypertension occurs earlier in men than women [19] and autoimmune diseases such as multiple sclerosis are more prevalent in women than men [20]. These comparisons will very likely uncover new drug targets for treating these devastating diseases, which is especially important given that we are now in an era where the drug discovery pipeline is shrinking; there were 50% fewer new drugs approved by the FDA and other major regulatory bodies between 2005–2010 than the previous five years [21].

Some individuals may argue that we are doing sufficient basic research in females when we carefully work out detailed mechanisms in males and then compare expression levels of one or two components in the elucidated male pathway to those in the female. But how can this extrapolation make biological sense when Kwekel et al. [10] have shown that whole gene networks crucial to biological pathways such as xenobiotic metabolism (cytochrome P450 enzymes and drug transporters) show major sexual dimorphic gene expression as a function of age across the life span? Sex-biased gene networks in female kidneys were over-represented in drug metabolism while male-biased gene networks were over-represented in renal dysfunction. These findings strongly argue that males and females exhibit distinct renal biology that is highly likely to impact the pharmacokinetics and pharmacodynamics of drug action. Given the inherent complexity of common chronic medical conditions, and the contributions of multiple genes to their etiology, it is imperative that we understand the full spectrum of gene networks within each sex and not simply extrapolate in a fragmented manner the function of one or two isolated genes in the female, as we now commonly do, from what we know mechanistically in-depth happens in the male (or vice-versa). Systems biology also cautions against this type of fragmented extrapolation when the goal is to understand complex traits. Systems biology research emphasizes the value of investigating principal networks of disease at the whole-body level and to integrate multiple processes like cell proliferation, metabolism and inflammation into complex disease settings like cardiovascular disease [22].

A major step to advancing healthcare requires the discovery of new cost-effective medicines; however, the productivity of the research and development (R&D) industry has been decreasing in recent years in part due to the enormous costs associated with drug discovery. Paul et al. [23] estimated a capitalized cost per launch of $1.8 billion using an R&D model that incorporates the probability of technical success, cost and cycle time starting from the point at which drugs are screened for specific drug targets (preclinical development) through Phase I, II and III/IV, the FDA approval process and the final launch of the new medicine. Their model, however, does not incorporate the cost of the basic biomedical research that serves as the foundation for identifying potential drug targets. Drug targets include biochemical compounds in the body such as DNA, RNA, proteins, glycans, lipids and small molecules, whose actions or absence of actions can result in disease processes. Most drug targets are discovered through basic biomedical research investigating physiological and pathophysiological mechanisms in biology, and the vast majority of funding for basic biomedical research comes from taxpayer dollars that are managed by the NIH.

## Conclusions

Given the remarkable sex differences that exist in disease including in the incidence, age of onset, symptoms, response to treatment and outcomes, isn’t it time to extend the basic principles embodied in Title IX that ban sex discrimination in education to also eliminate sex discrimination in biomedical research? Shouldn’t the movement started in this direction by the NIH revitalization Act of 1993 now include basic biomedical research? Can we afford to withdraw potential drugs at the final stages of drug development due to unforeseen adverse events in women, or to stop drug development along the way because a drug is less effective in one sex than the other? Can we afford to miss out on potential new drug targets by heavily biasing our drug discovery process towards male physiology and pathophysiology? Would a Title IX for basic biomedical research ensure that diminishing resources for research and R&D dollars are spent on discovering and developing drugs that optimally benefit both men and women, and reduce the investment losses of drugs that end up shelved, withdrawn or misused by physicians because of ineffectiveness or frequency of adverse events in one sex but not the other? A thoughtful, reasoned and evidence-based answer in 2013 can only be a resounding “Yes”!

## Abbreviations

FDA: U.S. Food and Drug Administration; GAO: U.S. General Accounting Office; NIH: National Institutes of Health; R&D: Research and Development.

## Competing interests

The authors have no competing interests.

## Authors’ contributions

KS conceived and wrote the article and JGV helped edit the manuscript. Both authors approved the final manuscript.

## References

[B1] United States Government Accounting Office Drug Safety: Most drugs withdrawn in recent years had greater health risks for women 2001 [http://www.gao.gov/new.items/d01286r.pdf]

[B2] United States Food and Drug Administration Public health advisory: Reports of valvular heart disease in patients receiving concomitant fenfluramine and phentermine 1997 [http://www.fda.gov/Drugs/DrugSafety/PostmarketDrugSafetyInformationforPatientsandProviders/DrugSafetyInformationforHeathcareProfessionals/PublicHealthAdvisories/ucm180072.htm]

[B3] Makkar RR Fromm BS Steinman RT Meissner MD Lehmann MH Female gender as a risk factor for torsades de pointes associated with cardiovascular drugs JAMA 1993 270 21 2590 2597 10.1001/jama.1993.03510210076031 8230644

[B4] United States Food and Drug Administration FDA Drug Safety Communication: Risk of next-morning impairment after use of insomnia drugs; FDA requires lower recommended doses for certain drugs containing zolpidem (Ambien, Ambien CR, Edluar, and Zolpimist) 2013 [http://www.fda.gov/Drugs/DrugSafety/ucm334033.htm]

[B5] United States Government Accounting Office Women’s Health: FDA needs to ensure more study of gender differences in prescription drug testing 1992 [http://archive.gao.gov/d35t11/147861.pdf]23905205

[B6] United States Food and Drug Administration Guidance for Industry: General considerations for the clinical evalulation of drugs in infants and children 1997 [http://www.fda.gov/downloads/Drugs/GuidanceComplianceRegulatoryInformation/Guidances/ucm071687.pdf]

[B7] Chen ML Lee SC Ng MJ Schuirmann DJ Lesko LJ Williams RL Pharmacokinetic analysis of bioequivalence trials: implications for sex-related issues in clinical pharmacology and biopharmaceutics Clin Pharmacol Ther 2000 68 5 510 521 10.1067/mcp.2000.111184 11103754

[B8] Pinnow E Sharma P Parekh A Gevorkian N Uhl K Increasing participation of women in early phase clinical trials approved by the FDA Womens Health Issues 2009 19 2 89 93 10.1016/j.whi.2008.09.009 19272558

[B9] Yang Y Carlin AS Faustino PJ Motta MI Hamad ML He R Watanuki Y Pinnow EE Khan MA Participation of women in clinical trials for new drugs approved by the food and drug administration in 2000–2002 J Womens Health (Larchmt) 2009 18 3 303 310 10.1089/jwh.2008.0971 19243271

[B10] Kwekel JC Desai VG Moland CL Vijay V Fuscoe JC Sex differences in kidney gene expression during the life cycle of F344 rats Biol Sex Differ 2013 in press 10.1186/2042-6410-4-14 PMC3844475 23902594

[B11] Yang X Schadt EE Wang S Wang H Arnold AP Ingram-Drake L Drake TA Lusis AJ Tissue-specific expression and regulation of sexually dimorphic genes in mice Genome Res 2006 16 8 995 1004 10.1101/gr.5217506 16825664 PMC1524872

[B12] van Nas A Guhathakurta D Wang SS Yehya N Horvath S Zhang B Ingram-Drake L Chaudhuri G Schadt EE Drake TA Elucidating the role of gonadal hormones in sexually dimorphic gene coexpression networks Endocrinology 2009 150 3 1235 1249 18974276 10.1210/en.2008-0563 PMC2654741

[B13] United States Food and Drug Administration Guidance for Industry: E16 biomarkers related to drug or biotechnology product development: context, structure, and format of qualification submissions 2011 [http://www.fda.gov/downloads/Drugs/GuidanceComplianceRegulatoryInformation/Guidances/UCM267449.pdf] 21834216

[B14] Ozer JS Dieterle F Troth S Perentes E Cordier A Verdes P Staedtler F Mahl A Grenet O Roth DR A panel of urinary biomarkers to monitor reversibility of renal injury and a serum marker with improved potential to assess renal function Nat Biotechnol 2010 28 5 486 494 10.1038/nbt.1627 20458319

[B15] Soldin OP Chung SH Mattison DR Sex differences in drug disposition J Biomed Biotechnol 2011 2011 1 14 10.1155/2011/187103 PMC3051160 21403873

[B16] Beery AK Zucker I Sex bias in neuroscience and biomedical research Neurosci Biobehav Rev 2011 35 3 565 572 10.1016/j.neubiorev.2010.07.002 20620164 PMC3008499

[B17] Maselli A Matarrese P Straface E Canu S Franconi F Malorni W Cell sex: a new look at cell fate studies FASEB J 2009 23 4 978 984 10.1096/fj.08-114348 19074513

[B18] United States Department of Justice Equal access to education: Forty years of Title IX 2012 [http://www.justice.gov/crt/about/edu/documents/titleixreport.pdf]

[B19] Sandberg K Ji H Sex differences in primary hypertension Biol Sex Differ 2012 3 7 1 21 22417477 10.1186/2042-6410-3-7 PMC3331829

[B20] Dunn SE Steinman L The gender gap in multiple sclerosis: intersection of science and society JAMA Neurol 2013 70 5 634 635 23460044 10.1001/jamaneurol.2013.55

[B21] Parexel International Corporation Parexel Biopharmaceutical R&D Statistical Sourcebook 2008 Waltham

[B22] MacLellan WR Wang Y Lusis AJ Systems-based approaches to cardiovascular disease Nat Rev Cardiol 2012 9 3 172 184 10.1038/nrcardio.2011.208 22231714 PMC3823242

[B23] Paul SM Mytelka DS Dunwiddie CT Persinger CC Munos BH Lindborg SR Schacht AL How to improve R&D productivity: the pharmaceutical industry’s grand challenge Nat Rev Drug Discov 2010 9 3 203 214 20168317 10.1038/nrd3078

